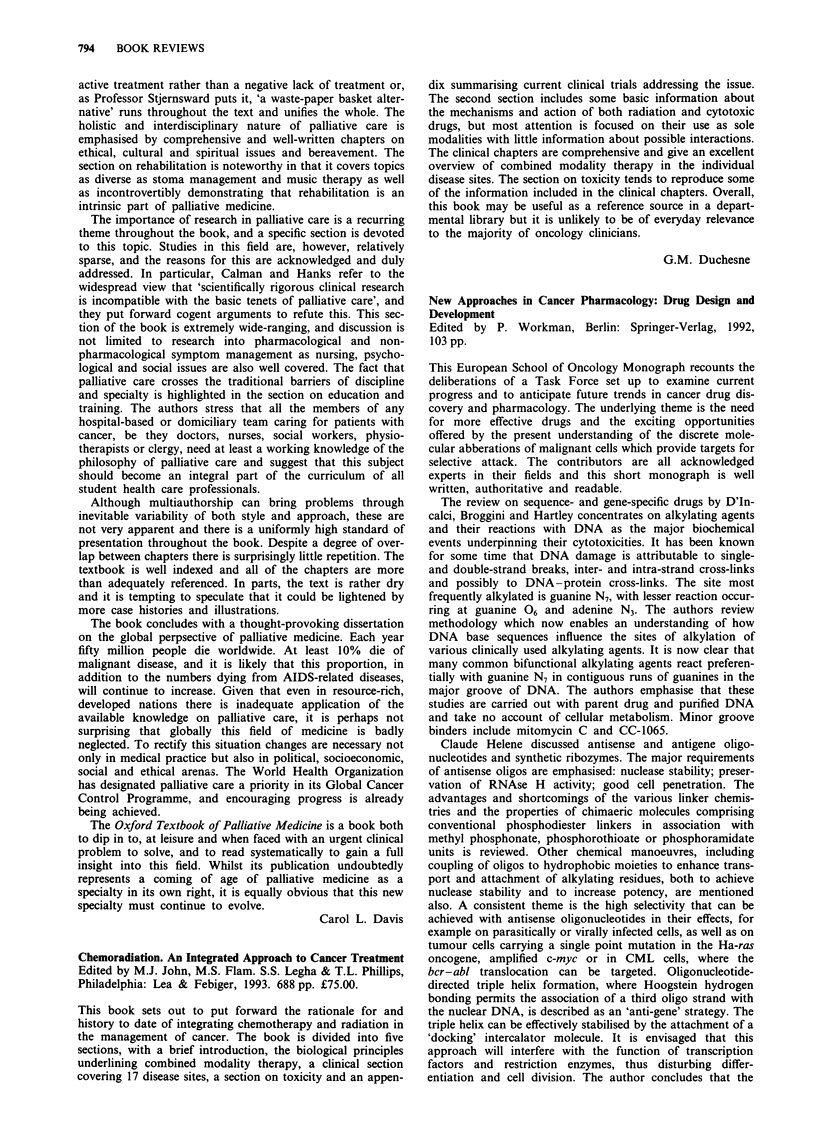# Chemoradiation. An Integrated Approach to Cancer Treatment

**Published:** 1994-04

**Authors:** G.M. Duchesne


**Chemoradiation. An Integrated Approach to Cancer Treatment**
Edited by M.J. John, M.S. Flam. S.S. Legha & T.L. Phillips, Philadelphia: Lea & Febiger, 1993. 688 pp. £75.00.

This book sets out to put forward the rationale for and history to date of integrating chemotherapy and radiation in the management of cancer. The book is divided into five sections, with a brief introduction, the biological principles underlining combined modality therapy, a clinical section covering 17 disease sites, a section on toxicity and an appendix summarising current clinical trials addressing the issue. The second section includes some basic information about the mechanisms and action of both radiation and cytotoxic drugs, but most attention is focused on their use as sole modalities with little information about possible interactions. The clinical chapters are comprehensive and give an excellent overview of combined modality therapy in the individual disease sites. The section on toxicity tends to reproduce some of the information included in the clinical chapters. Overall, this book may be useful as a reference source in a departmental library but it is unlikely to be of everyday relevance to the majority of oncology clinicians.

G.M. Duchesne